# Evening primrose oil ameliorates tissue architecture, apoptosis, and oxidative stress in the pancreas of diabetic rats: Possible role of miR-21

**DOI:** 10.22038/ijbms.2024.78604.17007

**Published:** 2024

**Authors:** Ali Louei Monfared, Ali Menatnia

**Affiliations:** 1 Department of Histology, Faculty of Veterinary Sciences, Ilam University, Ilam, Iran; 2 Graduate Student in Histology, Department of Histology, Faculty of Veterinary Sciences, Ilam University, Ilam, Iran

**Keywords:** Caspase-3, Diabetes, Evening primrose oil, Immunohistochemistry, Pancreas, Rat

## Abstract

**Objective(s):**

Activating apoptosis and oxidative stress contributes to the pathogenesis of diabetes. Evening primrose oil (EPO) has been shown to regulate lipid profiles and hyperglycemia under metabolic conditions. This study aimed to examine the effect of EPO on miR-21 expression, oxidative stress, apoptosis, and histological changes in the pancreas of male rats with experimental diabetes induced by streptozotocin (STZ).

**Materials and Methods:**

Thirty-two Wistar rats were divided into four distinct groups: control, diabetic, diabetic + EPO, and EPO. EPO was administered orally at a dose of 500 mg/kg, and STZ was administered intraperitoneally at a dose of 35 mg/kg for 28 days. In the end, the effects of treatments were assessed by measuring expressions of miR-21 in the pancreas with real-time PCR, pancreatic histological and immunohistochemical changes examinations, and oxidative stress assessment.

**Results:**

In the diabetic group, miR-21 expression and the levels of caspase-3 and malondialdehyde (MDA) were increased compared to the control group, while insulin expression and superoxide dismutase activity (SOD) levels were decreased significantly. Treatment with EPO resulted in a reduction of miR-21 and caspase-3 expression, as well as MDA levels, and an increase in insulin expression and SOD levels compared to the diabetic group. Additionally, supplementation with EPO demonstrated the ability to restore pancreatic tissue features, serum insulin levels, and blood glucose fluctuations.

**Conclusion:**

Collectively, the protective impacts of EPO in diabetic rats may be linked to the inhibition of miR-21/caspase-3/oxidative stress pathway, leading to the restoration of pancreatic β-cell function and structure.

## Introduction

Type 2 diabetes mellitus (T2DM) is a prevalent global health issue characterized by hyperglycemia (1). Chronic hyperglycemia can cause metabolic disturbances and sustained inflammation, thereby contributing to the development of various complications associated with T2DM (2). While genetic predisposition plays a role in the susceptibility to T2DM, environmental factors can also lead to an increased prevalence of the disease (3-5).

Studies have indicated that the diminished β-cell count in T2DM is believed to stem from the apoptosis of pancreatic β-cells. Elevated blood glucose levels associated with diabetes may disrupt the equilibrium between pro-apoptotic and anti-apoptotic factors, tipping it towards apoptosis and consequently promoting the demise of β-cells within pancreatic islets (6). Consequently, the apoptosis of pancreatic β-cells is considered a crucial factor in the onset and advancement of diabetes (7). 

MicroRNAs (miRNAs) are short and non-coding RNA molecules that are implicated in various cellular functions and metabolic diseases, including T2DM (8-10). Notably, microRNA-21 (miR-21) stands out as a particularly significant molecule potentially influencing the pathophysiology of T2DM and its complications (10, 11). For instance, the overexpression of miR-21 has been observed to play a crucial role in the development of diabetic retinopathy by contributing to diabetes-induced endothelial dysfunction and low-grade inflammation (12). In the renal tubules, miR-21 can regulate mesangial expansion, interstitial fibrosis, macrophage infiltration, podocyte loss, albuminuria, and the expression of fibrotic and inflammatory genes in streptozotocin-induced diabetic mice (13). MiR-21 exerts its influence on diabetic cardiomyopathy by impacting the proliferation and apoptosis of vascular smooth muscle cells, as well as the growth and death of cardiac cells and the functions of cardiac fibroblasts (14).

*Oenothera biennis, *commonly referred to as evening primrose, is a natural plant recognized for its medicinal properties. The oil derived from its seeds is notably abundant in omega-6 polyunsaturated fatty acids (PUFAs), including linoleic acid and gamma-linolenic acid (GLA). These PUFAs possess anti-inflammatory and anti-proliferative properties that contribute to the proper functioning of human tissues (15, 16). According to a prior report, evening primrose oil (EPO) in combination with vitamin D could reduce insulin resistance and improve hyperglycemia and lipid profiles in women with gestational diabetes (17). Furthermore, EPO and fish oil supplementations have shown potential in reducing hemoglobin A1c, fasting plasma glucose, total cholesterol, and body weight in individuals with non-insulin-dependent diabetes (18). Recently, the effect of EPO on adiponectin level and various biochemical parameters in a model of fructose-induced metabolic syndrome has been investigated. The findings suggest that EPO may serve as an antioxidant agent by mitigating oxidative stress, elevating adiponectin levels, and enhancing insulin sensitivity (19). A recent study indicated that EPO rich in GLA and fish oil is effective in ameliorating metabolic disorders related to obesity and diabetes mellitus (20). 

Despite the aforementioned research on the beneficial impacts of EPO on diabetes and its complications, the specific molecular and cellular mechanisms responsible for the restoration of pancreatic function and structure following EPO treatment remain elusive. Hence, the objective of this study is to evaluate the involvement of mir-21 in the ameliorative effects of EPO on the pancreas of diabetic rats, potentially by modulating the caspase-3/oxidative stress pathway.

## Materials and Methods

***Materials***

Streptozotocin (STZ) powder was acquired from Sigma-Aldrich Chemicals Co. (St. Louis, MO, USA). EPO was procured from Solgar, Inc. (Leonia, NJ, USA). Furthermore, the rat Insulin ELISA Kit (Cat No. # ER1113) was purchased from FineTest Company (Wuhan Fine Biotech Co, Ltd., China). An enzymatic-colorimetric glucose kit was purchased from Pars Azmoun Company (Glucose kit, Pars Azmoun Company, Tehran, Iran). Immunohistochemical staining was performed using primary monoclonal antibodies developed against Insulin (2D11-H5: sc-8033), cleaved caspase-3 p11 (h176-R: sc-22171-R) (Santa Cruz Biotechnology, SC-9038, Texas USA), Goat Anti-Mouse IgG(H+L)( Catalog No.# E-AB-1011; CY3 conjugated; Elabscience Biotechnology Inc. USA) and Goat Anti-Rabbit IgG(H+L) (Catalog No.# E-AB-1014 FITC conjugated; Elabscience Biotechnology Inc. USA). Determination of MicroRNA-21(miR-21) was done by using miRcute miRNA Isolation Kit (Catalog No. # DP501, Tiangen, Beijing, China) and miRcute miRNA First-strand cDNA Synthesis Kit (Catalog No. # KR201, TianGen, China). All other chemicals were obtained from Merck (Germany). 

***Animal husbandry and experimental design***

Thirty-two healthy adult male Wistar albino rats, aged ten weeks with an average weight of 190 ± 220 g, were utilized for the experimental trial. The rats were allowed to acclimatize to the new environment for one week before the commencement of treatments. The experimental design and animal handling protocols were approved by the Animal Ethics Committee at Ilam University in Ilam, Iran (Reference No.: IR.ILAM.REC.1401.014). The rats were divided into four groups, each comprising eight animals. The first group served as the control. The second group was induced T2DM by high-fat diet (HFD) feeding and low-dose STZ injection. The third group, labeled as T2DM + EPO, consisted of diabetic rats treated with EPO at a dose of 500 mg/kg (21). The fourth group comprised non-diabetic rats that also received EPO at the same dosage as the third group. All substances were administered to the rats daily via oral gavage. The administrations were repeated daily for four weeks.

***Induction of T2DM***

T2DM was induced in rats by utilizing a combination of an HFD and low-dose STZ according to the methodology described by Daneshyar *et al*. (2014) (22). Rats in the non-diabetic control group were administered an equal volume of sterile 0.9% saline solution, and the diabetic groups were subjected to an HFD for four weeks, followed by the intraperitoneal administration of a low dose of STZ (35 mg/kg) in a 0.1 M citrate buffer with a pH of 4.5. Diabetic rats were identified three days post-injection based on non-fasting serum glucose levels of ≥300 mg/dl obtained from the tail vein and subsequently included in further investigations.

***Blood and tissue sampling***

Rats in each experimental group were weighed at the beginning and end of the study period to determine body weight gain by calculating the difference between initial and final weights. For biochemical examinations, blood samples were collected from all rat groups after four weeks of treatment. The serum was then isolated by centrifugation (10 min, 3000 rpm, at 4 ºC) and stored at -20 °C for subsequent biochemical analysis. Pancreatic tissues were promptly collected and divided into two parts. The first part was stored at -80 °C for further evaluation of antioxidant status and miRNA levels in tissue homogenate. The second part was fixed in neutral buffered formaldehyde at 4 ºC for two days to facilitate future tissue analyses.

***Assessment of the fasting blood glucose (FBG) and insulin*** ***levels ***

FBG levels were assessed through colorimetric measurement using an enzyme technique with glucose oxidase (23). Serum insulin levels were quantified using a rat-specific enzyme-linked immunosorbent assay (ELISA) kit following the manufacturer’s instructions (23).

***Quantification of oxidative/anti-oxidative biomarkers***

The estimation of malondialdehyde (MDA) in the tissue was estimated using the method of Mihara and Uchiyama in 1978 (24). Briefly, 50 microliters of the homogenized tissue sample were mixed with 250 microliters of a solution containing 20% trichloroacetic acid and 100 microliters of 0.6% thiobarbituric acid and heated for at least 20 min in a boiling water bath. Then, the samples were cooled and centrifuged at 5000 rpm for 5 min to remove impurities and make the supernatant clear. Then, from each sample, 200 microliters of the supernatant were transferred to a 96-well plate, and the absorption of the samples against the blank was read at a wavelength of 535 nm by a spectrophotometer (Bio-Tek, Winooski, VT, USA). Superoxide dismutase activity (SOD) in the tissues was assayed by the method of Nishikimi *et al*. in 1972 (25). In brief, 0.5 ml of homogenized tissue sample was diluted with 1 ml of water. Then, 2.5 ml of ethanol and 1.5 ml of chloroform were added and shaken for one minute at 4 °C then centrifuged. The enzyme activity in the supernatant was determined. The reaction mixture contained 0.53g of sodium carbonate buffer (50 mM, pH=10.2) in 100 ml dH2o, 0.003 gr of EDTA (0.1 mM), 30µl of 0.03% triton-X-100, 0.002 gr of nitro blue tetrazolium (NBT, 24 µM) and 0.007 gr of Hydroxylamine hydrochloride (1 mM), appropriately diluted enzyme preparation and water. The absorption of the samples against the blank was read at a wavelength of 560 nm. The activity of catalase (CAT) in the tissues was measured using the 1952 method of Beers and Sizer (26). The reaction mixture contained phosphate buffer (50 mM, pH 7.4), pancreas tissue homogenate (10 µl), and H_2_O_2_ (20 mM). Absorbance changing in the tissues was determined after the reduction of H_2_O_2_ at 240 nm for 10 min.

***Determination of microRNA-21(miR-21)***

Total RNA was extracted from 50 mg of pancreas tissue by using the miRcute miRNA Isolation Kit (Tiangen, Beijing, China), according to the supplier’s protocol. The purity of RNA was determined by measuring the optical density at a 260/280 OD ratio using an Eppendorf µCuvette G1.0 micro-volume measuring cell (Eppendorf, Germany). Purified RNA from each sample containing 300 ng of total RNA, was promptly reverse-transcribed into first-strand cDNA using miRcute miRNA First-strand cDNA Synthesis Kit (TianGen, China). Subsequently, qRT-PCR was performed using the ABI Stepone Plus detection system (ABI, USA) by the miRcute miRNA qPCR Detection Kit, SYBR Green (TianGen, China) and a specific primer for miR-21. The reaction was conducted under the following conditions (94 °C for 2 min, 94 °C for 20 sec, and 60 °C for 34 sec. The relative quantity of miR-21 for each sample was normalized to the U_6_ level as the endogenous control (27). The delta-delta-cycle threshold values were computed, and then the 2-(▵▵^Ct^) method and Stepone software 2.3 were employed to determine the relative quantitative levels of miR-21. The primer sequences for miR-21 forward were: AGCTTATCAGACTGATGTTG; cDNA adapter reverse was: GAACATGTCTGCGTATCTC; U_6_ Forward was: CTCGCTTCGGCAGCACA; U6-Reverse was: AACGCTTCACGAATTTGCGT. Sequences were retrieved from GenBank. Primer specificity was confirmed using Gene Runner software (Syngene) and validated by Oligo 7 software.

***Histological examination***

The pancreatic tissue samples were preserved in neutral buffered formaldehyde at 4 °C for two days for microscopic analysis. Following this, the tissues underwent standard processing steps, including dehydration in ethanol, clearing in xylene, and embedding in paraffin. Sections of 5 μm thickness were then cut using a microtome and stained with H & E. The evaluation of pancreatic changes involved quantifying the perimeter of pancreatic islets according to the method described by Faried and El-Mehi (28). Furthermore, the number of β‐cells within each islet was counted under a 40x magnification, and cell densities were normalized per pancreatic islet using the approach outlined by Wang-Fischer and Garyantes (29). For each sample (from 5 different rats per group), five random fields (40×) were chosen using a Leica microscope equipped with a digital camera and True Chrome Metrics software (China) to analyze each parameter.

***Immunohistochemistry (IHC)***

Pancreas tissue sections were rinsed in phosphate buffer, and endogenous peroxidases were neutralized with a solution of 3% hydrogen peroxide in 50% ethanol for 15 min. Subsequently, a microwave-based antigen retrieval technique was employed. The sections were then blocked using 5% normal goat serum and subjected to overnight incubation with primary antibodies at a temperature of 4°C. The primary antibodies utilized in this study were anti-insulin (dilution ratio of 1:100) and anti-caspase-3 (dilution ratio of 1:100) (30). Following this, the slides were washed with phosphate buffer saline and exposed to a fluorescent dye-conjugated goat secondary antibody (dilution ratio of 1:150) for one hour. Diaminobenzidine was employed for color development on the tissue sections, and counter-staining was performed using H & E solution. Post-counter-staining with DAPI at 4 °C for 10 min, positive signals were visualized under a fluorescent microscope (Olympus BX50), and images were captured using a digital camera (Olympus DP72). In the evaluation of protein expression via IHC, five random fields were selected from each sample for analysis. A semi-quantitative protein IHC intensity score was then assigned based on the percentage of the protein-positive area using ImageJ software. The results are presented relative to the control group. Additionally, the percentage of the area showing immunoreactivity for insulin and caspase-3 was quantified (28).

***Statistical analysis***

Statistical analysis was conducted utilizing SPSS software (IBM, Armonk, NY, USA). The data were expressed as mean ± standard error (M± SE). The Shapiro-Wilk test was used to verify normality, and the Kruskal-Wallis test was used to measure negative data in the normality test. The data were subjected to statistical analysis through one-way analysis of variance, followed by Tukey’s *post-hoc* test to assess intergroup differences in the parameters. Statistical significance was defined as *P*<0.05.

## Results

***Effect of EPO treatment on insulin, FBG, and body weight gain***

Table 1 shows that the insulin levels in diabetic rats decreased significantly compared to the control group. However, administration of EPO to diabetic rats increased their insulin levels compared to the untreated diabetic group, although it remained lower than that of the control group. Diabetic rats also displayed a notable increase in FBG levels compared to the control group. Following a four-week EPO treatment in diabetic rats, FBS levels decreased to levels akin to those of the control group. Initially, the average body weights among the various groups were similar. By the end of the treatment period, the final body weights of diabetic rats had significantly decreased compared to the control rats. However, treatment of diabetic rats with EPO led to a non-significant increase in final body weights when compared to the untreated diabetic group, as indicated in Table 1.

***Effect of EPO treatment on oxidative stress biomarkers***

The levels of MDA, SOD, and CAT in pancreatic tissue among the four experimental groups are presented in Table 2. Diabetic animals exhibited significantly higher MDA levels compared to both the control and EPO groups. Conversely, diabetic rats treated with EPO for four weeks showed a notable reduction in MDA levels in pancreatic tissue compared to untreated diabetic rats. Additionally, diabetic rats demonstrated a significant decrease in pancreatic SOD activity compared to the control and EPO groups. In contrast, treatment of diabetic rats with EPO resulted in a significant increase in SOD concentration compared to the diabetic group (Table 2). Evaluation of CAT levels in pancreatic homogenates from diabetic rats indicated a significant decrease compared to the control group. There was no significant difference in CAT levels between diabetic rats treated with EPO and untreated diabetic control rats during EPO treatment. Notably, EPO treatment did not significantly alter CAT levels in diabetic animals (Table 2).

***Effect of EPO treatment on miR-21expression***

**Figure 1:**
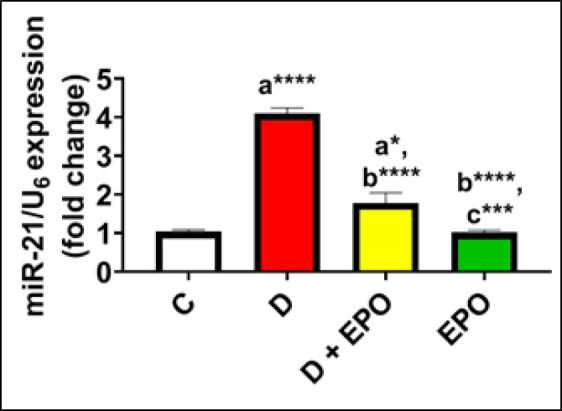
Effect of EPO on the pancreatic expression of miR-21 in diabetic rats

The data presented in Figure 1 demonstrates that miR-21 expression was notably elevated in diabetic rats when compared to the control group. Moreover, diabetic rats treated with EPO exhibited a decreased fold change in miR-21 expression in comparison to untreated diabetic rats, although it remained higher than that of the control rats. Furthermore, a significant decrease in the fold change of miR-21 expression was noted in the EPO-treated group when compared to the diabetic group, as depicted in Figure 1.

***Effect of EPO treatment on pancreas histology and histomorphometry***

**Figure 2:**
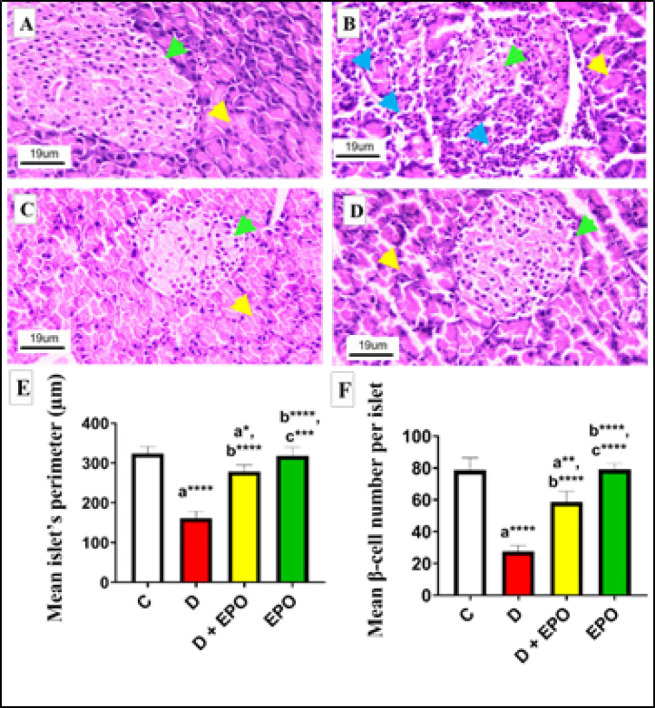
Effect of EPO on the pancreas architecture-related parameters (H&E)

The histological changes in rat pancreas for all groups are shown in Figure 2. Pancreatic tissue samples from the control group exhibited normal appearance of islets characterized by a high concentration of β-cells. Conversely, the islets in the diabetic group appeared diminished in size and were infiltrated with mononuclear leukocytes, rendering them challenging to discern. Conversely, the diabetic group treated with EPO displayed a notable enhancement in islet morphology, with evident mitigation of most degenerative and infiltrative changes (Figure 2-C). Morphometric results (Figure 2-E-F) indicated a significant decrease in islet perimeter and β-cell density/islets of diabetic rats compared to those in the control group. In contrast, treatment with EPO in diabetic rats led to a substantial increase in islet perimeter and β-cell count compared to the diabetic group.

***Effect of EPO treatment on the expressions of insulin and caspase-3***

**Figure 3:**
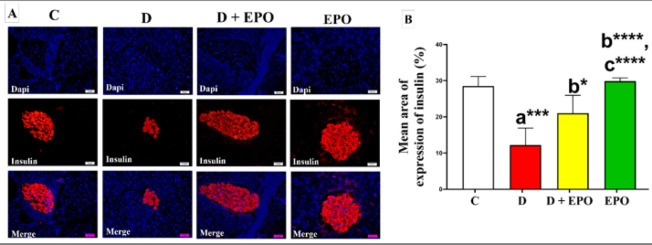
Effect of EPO on the immunohistochemical expression of anti-insulin antibody in diabetic rats (Immunoperoxidase X400)

As shown in Figures 3-A and 3-B, pancreatic sections of the control group revealed consistently positive immunoreactivity against insulin antibodies. The pancreatic tissue sections of diabetic rats displayed a notable decrease in insulin immunoreactivity in comparison to the control group. Conversely, the diabetic rats treated with EPO exhibited a significant rise in insulin antigen positivity within the majority of β-cells, as illustrated in Figure 3-A.

**Figure 4:**
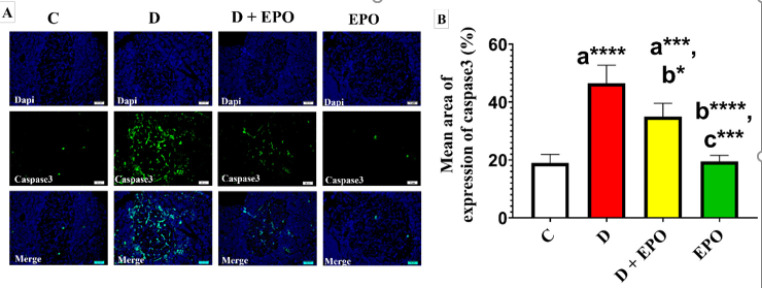
Effect of EPO on the immunohistochemical expression of anti- caspase-3 antibody in diabetic rats (Immunoperoxidase X400)

As Figure 4-A-B shows a significant increase in islets immunoreactivity against caspase-3 antibody was noted in the diabetic group compared to the control group. Conversely, the diabetic rats treated with EPO showed a considerable reduction in caspase-3 immunoreactivity compared to the diabetic group (Figure 4). 

## Discussion

Diabetes impacts health globally, with millions affected by its various forms. It can lead to complications such as heart disease, kidney failure, nerve damage, and vision loss if not managed properly (31). Diabetes impairs the function of the pancreas, especially in type 1 diabetes, where the pancreas is unable to produce insulin, or in type 2 diabetes, the pancreas may not produce enough insulin, or the body may become resistant to its effects (32). Both scenarios can lead to disruptions in blood sugar regulation and overall pancreatic health. Since EPO is receiving increasing attention for its potential in human medicine, the purpose of this study was to investigate its efficiency toward diabetes-induced pancreas damage in male Wistar rats. Our results reveal that T2DM causes severe lesions to the pancreatic architecture that were accompanied by a higher expression of miR-21, MDA, and disturbance of the glucose/insulin profile in male rats. Inversely, EPO supplementation could significantly promote histological and functional recoveries of the pancreas. To the best of our knowledge, this study is the first experimental work examining the association between EPO treatment with pancreatic tissue expression of miR-21, oxidative stress, and apoptosis in the T2DM model induced by HFD feeding and STZ injection. 

Based on the current results, there was a notable decrease in body weight observed in the untreated diabetic group. This observation aligns with the findings of Metawea *et al*. (33), who also documented weight loss in diabetic rats. The diminished body weight in diabetic rats is likely attributed to factors such as tissue protein breakdown (34), reduced glucose metabolism, and heightened fat metabolism (35). Surprisingly, the administration of EPO could not restore the weight loss caused by diabetes. The precise mechanism for EPO’s insufficiency in compensating for T2DM-induced weight loss is most likely due to its active fatty acids ingredient, GLA. In this regard, it has been demonstrated that higher levels of GLA in the serum are significantly and inversely associated with weight gain in preschool children (36). On the other hand, the involvement of GLA in the suppression of fat accretion in obese adults has been investigated (37). 

**Table 1:** Effect of EPO on the values of fasting insulin, fasting blood glucose, and body weight gain of diabetic rats

Parameters	C	D	D + EPO	EPO
Insulin (pmol/l)	83 ± 1.4	32.8± 1.9^ a****^	63.6 ± 5.7^ a**,b****^	87 ± 0.71^ b****^
FBG (mg/dl)	94.00 ± 0.84	420.8 ± 5.07^ a****^	105.2 ±1.59^ b****^	81.8 ±1.39^ a*,b****^
Initial body weight (g)	196.5 ± 4.39	195 ± 2.46	194 ±3.12	195.2 ±4.31
Final body weight (g)	234.8 ± 1.2	199.6 ± 0.52^ a****^	206.4 ±1.03^ a****^	229 ±2.37

Each row represents M± SE (n=8). The letters ^a ^and ^b^ indicate significant differences when compared with the control and diabetes groups, respectively. *P<0.05, **P<0.01, and ****P<0.0001. C: Control rats; D: Diabetic rats; D + EPO: Diabetic rats that were given EPO; EPO: Non-diabetic rats subjected to EPO; FBG: Fasting blood glucose

**Table 2:** Effect of EPO on the values of oxidative stress biomarkers of diabetic rats

Parameters	C	D	D + EPO	EPO
MDA (nmol/ml)	2.64 ± 0.18	7.24± 0.45^ a****^	4 ± 0.13^ a*,b****^	2.33 ± 0.22^ b****, c**^
SOD (U/ml)	69.5 ± 2.8	35.4 ± 3.1^ a****^	52.03 ±4.9^ a*, b*^	68.6 ±2.2^ b****, c*^
CAT (U/ml)	15.49 ± 0.92	6.59 ± 0.68^ a***^	9.33 ±0.24^ a*^	16.05 ±1.47^ b***, c**^

Each row represents M± SE (n=8). The letters a, b, and c indicate significant differences when compared with the control, diabetes, and diabetic plus EPO groups, respectively. *P<0.05, **P<0.01, ***P<0.001, and ****P<0.0001. C: Control rats; D: Diabetic rats; D + EPO: Diabetic rats that were given EPO; EPO: Non-diabetic rats subjected to; MDA: Malondialdehyde; SOD: Superoxide dismutase; CAT: Catalase

Data were expressed as the M± SE (n=8). The letters ^a^,^ b^, and ^c^ on the bars indicate significant differences when compared with the control, diabetes, and diabetic + EPO rat groups, respectively. *P<0.05, ***P<0.001, and ****P<0.0001. C: Control rats; D: Diabetic rats; D + EPO: Diabetic rats given EPO; EPO: Non-diabetic rats subjected to EPO.

(A) the control group; (B) the diabetic group; (C) diabetic rats treated with EPO; and (D) non-diabetic rats treated with 500 mg/kg EPO, with 400× magnification; the green, yellow, and blue arrows represent islets, exocrine acini, and leukocytes infiltration, respectively. (E-F): Effect of EPO on the islet perimeter (E) and β-cell’s number (F) in pancreatic tissue sections of diabetic rats. The letters of a, b, and c on the bars indicate significant differences when compared with the control, diabetes, and diabetic + EPO rat groups, respectively. *P<0.05, **P<0.01, ***P<0.001, and ****P<0.0001. C: Control rats; D: Diabetic rats; D + EPO: Diabetic rats given EPO; EPO: Non-diabetic rats subjected to EPO.

(A): Representative fluorescent images of pancreatic tissue sections from the different studied groups showing immunohistochemical detection of Dapi (blue), anti-insulin antibody (red), and merged images of insulin and Dapi (blue and red) in islet cells. Scale bar, 20 µm. (B): Quantitative analysis of insulin expression in the islet area. Scale bar, 20 µm. The letters of ^a^, ^b^, and ^c ^on the bars indicate significant differences when compared with the control, diabetes, and diabetic + EPO rat groups, respectively. *P<0.05, ***P<0.001, and ****P<0.0001. C: Control rats; D: Diabetic rats; D + EPO: Diabetic rats given EPO; EPO: Non-diabetic rats subjected to EPO.

(A): Representative fluorescent images of pancreatic tissue sections from the different studied groups showing immunohistochemical detection of Dapi (blue), anti-caspase-3 antibody (green), and merged images of caspase-3 and Dapi (green and red) in islet cells. (B): Quantitative analysis of caspase-3 expression in the islet area. Scale bar, 20 µm. The letters ^a^, ^b^, and^ c^ on the bars indicate significant differences when compared with the control, diabetes, and diabetic + EPO rat groups, respectively. *P<0.05,***P<0.001, and ****P<0.0001. C: Control rats; D: Diabetic rats; D + EPO: Diabetic rats given EPO; EPO: Non-diabetic rats subjected to EPO.

**Figure 5:**
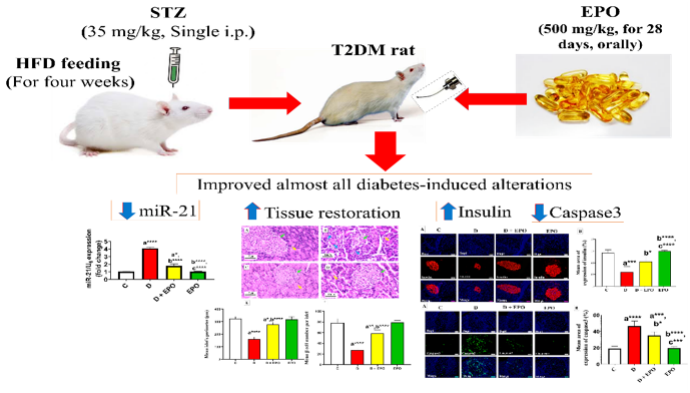
A proposed schematic diagram for the ameliorative effect of EPO on the pancreas of high-fat diet and low-dose streptozotocin-induced type 2 diabetes rat model

EPO: Non-diabetic rats subjected to EPO

In the current investigation, rats with diabetes induced by an HFD and STZ exhibited a notable rise in blood glucose levels and a substantial reduction in serum insulin concentrations. Conversely, the administration of EPO to diabetic rats resulted in a significant improvement in glycemic control. Consistent with our results, a prior study has demonstrated that EPO oil rich in GLA can enhance insulin sensitivity in mouse tissues, thereby reducing serum glucose levels. Furthermore, both GLA oil and fish oil have been shown to enhance serum insulin levels, consequently eliciting hypoglycemic effects (20).

The progression of T2DM has been strongly linked to oxidative stress (38). It is also theorized that hyperglycemia triggers oxidative stress, resulting in tissue damage from the oxidation of biomolecules (39,40). Our study on diabetic rats revealed an elevation in MDA levels and a significant reduction in SOD levels. Notably, the administration of EPO significantly suppressed the activation of MDA in the pancreas of T2DM-induced rats while also enhancing SOD levels. This suggests that EPO, being a rich source of GLA, may act by mitigating oxidative stress to repair pancreatic damage caused by T2DM. These results are consistent with a previous study that highlighted the antioxidant properties of EPO supplementation in countering metanil yellow-induced hepatotoxicity in rats(41). Moreover, our microscopic observations revealed enhancements in infiltrative alterations, expansion of islet perimeter, and an increase in the number of β-cells. These results suggest that EPO exhibits anti-inflammatory properties against β-cell dysfunction induced by oxidative stress.

The findings of our study revealed up-regulation of miR-21 expression in the pancreas of rats with T2DM. This molecular alteration was accompanied by a diminished average area occupied by insulin immunoreactivity in immunohistochemical experiments. These results align with previous research that has reported elevated levels of miR-21-3p in both T2DM and pre-T2DM individuals (11, 42). Conversely, administration of EPO to diabetic rats led to a reduction in miR-21 expression in pancreatic tissue and a decrease in caspase-3 levels, a marker of apoptosis. These results indicate that the reduction in pancreatic caspase-3 expression may be mediated by the decrease in miR-21 levels following the administration of EPO. The beneficial effects of EPO are likely attributed to its bioactive components, including GLA, phenolic acids, flavonoids, and herbal sterols (43). Furthermore, PUFAs have been shown to modulate the expression of miRNAs, such as miR-21, in a time- and dose-dependent manner (44). Therefore, the observed protective role of EPO against T2DM in our study may involve the suppression of miR-21 activity, thereby preventing caspase-3-mediated apoptosis of pancreatic β-cells.

The discovery of novel molecular targets associated with the progression of T2DM holds significant scientific importance. Previous research has demonstrated the role of miRNAs in regulating the apoptosis process(9). MiR-21 has been identified as a pathogenic factor that promotes apoptosis by targeting forkhead box O1 (FOXO1) in podocyte injury induced by high glucose (45, 46). Overexpression of miR-21 has been shown to reduce cell viability and increase levels of cleaved caspase-3, indicating enhanced cell death (47). The current study revealed a significant increase in caspase-3-mediated β-cell apoptosis in the diabetic group compared to the control group, accompanied by a reduction in islet perimeter and β-cell count, consistent with previous research (28). These findings suggest that up-regulation of miR-21 in pancreatic tissue may contribute to β-cell apoptosis in the hyperglycemic state. Conversely, treatment with EPO in diabetic rats led to a notable decrease in caspase-3 immunoreactivity compared to untreated diabetic rats, indicating the anti-apoptotic effects of EPO and supporting pancreatic recovery. There is currently a lack of data regarding the impact of EPO on the expression levels of miR-21 in the pancreatic tissue of diabetic rats.

Our findings indicate a significant association between diabetes, caspase-3 immunoreactivity, mir-21 overexpression, and dysfunction of β-cells in the pancreatic tissue of diabetic rats. However, several limitations should be acknowledged. The study did not assess the impact of EPO on inflammatory markers. Additionally, the absence of a luciferase reporter assay, a valuable tool for identifying and analyzing potential target genes associated with miR-21, was another constraint of this research. Due to constraints related to time and funding, only light microscopy examinations were conducted instead of electron microscopic ultrastructural studies. Consequently, the precise relationship between EPO and miR-21 necessitates further investigation in subsequent studies.

## Conclusion

These findings indicate the role of EPO in ameliorating the pancreas β-cells integrity in rats suffering from T2DM. The protective effects of EPO against oxidative stress and apoptosis in rats with T2DM may be linked to the inhibition of miR-21. This inhibition leads to the restoration of the function and morphology of pancreatic β-cells (Figure 5).

## Data Availability

All data are fully available without restriction.

## References

[1] Al-Attar AM, Alsalmi FA (2019). Effect of Olea europaea leaves extract on streptozotocin induced diabetes in male albino rats. Saudi J Biol Sci.

[2] Rohm TV, Meier DT, Olefsky JM, Donath MY (2022). Inflammation in obesity, diabetes, and related disorders. Immunity.

[3] Ajlouni K, Batieha A, Jaddou H, Khader Y, Abdo N, El‐Khateeb M (2019). Time trends in diabetes mellitus in Jordan between 1994 and 2017. Diabet Med.

[4] Khader YS, Batieha A, Jaddou H, Batieha Z, El-Khateeb M, Ajlouni K (2011). Relationship between 25-hydroxyvitamin D and metabolic syndrome among Jordanian adults. Nutr Res Pract.

[5] Magkos F, Hjorth MF, Astrup A (2020). Diet and exercise in the prevention and treatment of type 2 diabetes mellitus. Nat Rev Endocrinol.

[6] Federici M, Hribal M, Perego L, Ranalli M, Caradonna Z, Perego C (2001). High glucose causes apoptosis in cultured human pancreatic islets of Langerhans: A potential role for regulation of specific Bcl family genes toward an apoptotic cell death program. Diabetes.

[7] Khin PP, Lee JH, Jun H-S (2023). Pancreatic beta-cell dysfunction in type 2 diabetes. Eur J Inflamm.

[8] Özcan S (2015). MicroRNAs in pancreatic β-cell physiology. Adv Exp Med Biol.

[9] Jovanovic M, Hengartner M (2006). MiRNAs and apoptosis: RNAs to die for. Oncogene..

[10] Liu R, Liu C, He X, Sun P, Zhang B, Yang H (2022). MicroRNA-21 promotes pancreatic β cell function through modulating glucose uptake. Nat Commun.

[11] Olivieri F, Spazzafumo L, Bonafè M, Recchioni R, Prattichizzo F, Marcheselli F (2015). MiR-21-5p and miR-126a-3p levels in plasma and circulating angiogenic cells: Relationship with type 2 diabetes complications. Oncotarget.

[12] Roy D, Modi A, Khokhar M, Sankanagoudar S, Yadav D, Sharma S (2021). MicroRNA 21 emerging role in diabetic complications: A critical update. Curr Diabetes Rev.

[13] Kölling M, Kaucsar T, Schauerte C, Hübner A, Dettling A, Park JK (2017). Therapeutic miR-21 silencing ameliorates diabetic kidney disease in mice. Mol Ther.

[14] Cheng Y, Zhang C (2010). MicroRNA-21 in cardiovascular disease. J Cardiovasc Transl Res.

[15] Timoszuk M, Bielawska K, Skrzydlewska E (2018). Evening primrose (Oenothera biennis) biological activity dependent on chemical composition. Antioxidants.

[16] Taweechaisupapong S, Srisuk N, Nimitpornsuko C, Vattraphoudes T, Rattanayatikul C, Godfrey K (2005). Evening primrose oil effects on osteoclasts during tooth movement. Angle Orthod.

[17] Jamilian M, Karamali M, Taghizadeh M, Sharifi N, Jafari Z, Memarzadeh MR (2016). Vitamin D and evening primrose oil administration improve glycemia and lipid profiles in women with gestational diabetes. Lipids.

[18] Takahashi R, Inoue J, Ito H, Hibino H (1993). Evening primrose oil and fish oil in non-insulin-dependent-diabetes. Prostaglandins Leukot Essent Fatty Acids.

[19] Mert H, İrak K, Çibuk S, Yıldırım S, Mert N (2022). The effect of evening primrose oil (Oenothera biennis) on the level of adiponectin and some biochemical parameters in rats with fructose induced metabolic syndrome. Arch Physiol Biochem.

[20] Ide T (2023). γ-Linolenic Acid-rich oil-and fish oil-induced alterations of hepatic lipogenesis, fatty acid oxidation, and adipose tissue mRNA expression in obese KK-A y mice. J Oleo Sci.

[21] Kaya Z, Eraslan G (2013). The effects of evening primrose oil on arsenic-induced oxidative stress in rats. Toxicol Environ Chem.

[22] Daneshyar S, Gharakhanlou R, Nikooie R, Forutan Y (2014). The effect of high-fat diet and streptozotocin-induced diabetes and endurance training on plasma levels of calcitonin gene-related peptide and lactate in rats. Can J Diabetes.

[23] Gherbon A, Frandes M, Dîrpeş D, Timar R, Timar B (2024). Impact of SGLT-2 inhibitors on modifiable cardiovascular risk factors in Romanian patients with type 2 diabetes mellitus. Diabetol Metab Syndr.

[24] Mihara M, Uchiyama M (1978). Determination of malonaldehyde precursor in tissues by thiobarbituric acid test. Anal Biochem.

[25] Nishikimi M, Appaji N, Yagi K (1972). The occurrence of superoxide anion in the reaction of reduced phenazine methosulfate and molecular oxygen. Biochem Biophys Res Commun.

[26] Beers rf Jr, Sizer Iw (1952). A spectrophotometric method for measuring the breakdown of hydrogen peroxide by catalase. J Biol Chem.

[27] Chen LY, Wang X, Qu XL, Pan LN, Wang ZY, Lu YH (2019). Activation of the STAT3/microRNA-21 pathway participates in angiotensin II-induced angiogenesis. J Cell Physiol.

[28] Faried MA, El-Mehi AE-S (2020). Aqueous anise extract alleviated the pancreatic changes in streptozotocin-induced diabetic rat model via modulation of hyperglycaemia, oxidative stress, apoptosis and autophagy: A biochemical, histological and immunohistochemical study. Folia Morphologica.

[29] Wang-Fischer Y, Garyantes T (2018). Improving the reliability and utility of streptozotocin-induced rat diabetic model. J Diabetes Res.

[30] Tomita T (2010). Immunocytochemical localization of cleaved caspase-3 in pancreatic islets from type 1 diabetic subjects. Islets.

[31] Razaq RA, Mahdi JA, Jawad RA (2020). Information about diabetes mellitus. J Univ Babylon Pure Appl Sci.

[32] Radlinger B, Ramoser G, Kaser S (2020). Exocrine pancreatic insufficiency in type 1 and type 2 diabetes. Curr Diab Rep.

[33] Metawea MR, Abdelrazek HM, El-Hak HNG, Moghazee MM, Marie OM (2023). Comparative effects of curcumin versus nano-curcumin on histological, immunohistochemical expression, histomorphometric, and biochemical changes to pancreatic beta cells and lipid profile of streptozocin induced diabetes in male Sprague–Dawley rats. Environ Sci Pollut Res.

[34] Yanardag R, Ozsoy-Sacan O, Bolkent S, Orak H, Karabulut-Bulan O (2005). Protective effects of metformin treatment on the liver injury of streptozotocin-diabetic rats. Hum Exp Toxicol.

[35] Rossmeisl M, Rim JS, Koza RA, Kozak LP (2003). Variation in type 2 diabetes-related traits in mouse strains susceptible to diet-induced obesity. Diabetes.

[36] Perng W, Villamor E, Mora-Plazas M, Marin C, Baylin A (2015). Alpha-linolenic acid (ALA) is inversely related to development of adiposity in school-age children. Eur J Clin Nutr.

[37] Schirmer MA, Phinney SD (2007). γ-Linolenate reduces weight regain in formerly obese humans. J Nutr.

[38] Abdulmalek S, Eldala A, Awad D, Balbaa M (2021). Ameliorative effect of curcumin and zinc oxide nanoparticles on multiple mechanisms in obese rats with induced type 2 diabetes. Sci Rep.

[39] Cecilia O-M, José Alberto C-G, José N-P, Ernesto Germán C-M, Ana Karen L-C, Luis Miguel R-P (2019). Oxidative stress as the main target in diabetic retinopathy pathophysiology. J Diabetes Res.

[40] Albert-Garay JS, Riesgo-Escovar JR, Salceda R (2022). High glucose concentrations induce oxidative stress by inhibiting Nrf2 expression in rat Müller retinal cells in vitro. Sci Rep.

[41] Shalaby AM, Shalaby RH, Alabiad MA, Abdelrahman DI, Alorini M, Jaber FA (2023). Evening primrose oil attenuates oxidative stress, inflammation, fibrosis, apoptosis, and ultrastructural alterations induced by metanil yellow in the liver of rat: A histological, immunohistochemical, and biochemical study. Ultrastruct Pathol.

[42] Yazdanpanah Z, Kazemipour N, Kalantar SM, Vahidi Mehrjardi MY (2022). Plasma miR‐21 as a potential predictor in prediabetic individuals with a positive family history of type 2 diabetes mellitus. Physiol Rep.

[43] Mohammad HM, El-Baz AA, Mahmoud OM, Khalil S, Atta R, Imbaby S (2023). Protective effects of evening primrose oil on behavioral activities, nigral microglia and histopathological changes in a rat model of rotenone-induced parkinsonism. J Chem Neuroanat.

[44] LeMay-Nedjelski L, Mason-Ennis JK, Taibi A, Comelli EM, Thompson LU (2018). Omega-3 polyunsaturated fatty acids time-dependently reduce cell viability and oncogenic microRNA-21 expression in estrogen receptor-positive breast cancer cells (MCF-7). Int J Mol Sci.

[45] Wang J, Shen L, Hong H, Li J, Wang H, Li X (2019). Atrasentan alleviates high glucose-induced podocyte injury by the microRNA-21/forkhead box O1 axis. Eur J Pharmacol.

[46] Liu L, Wang Y, Yan R, Liang L, Zhou X, Liu H (2019). BMP-7 inhibits renal fibrosis in diabetic nephropathy via miR-21 downregulation. Life Sci.

[47] Sims EK, Lakhter AJ, Anderson-Baucum E, Kono T, Tong X, Evans-Molina C (2017). MicroRNA 21 targets BCL2 mRNA to increase apoptosis in rat and human beta cells. Diabetologia.

